# Exploring the Perceptions of Voice-Assisted Technology as a Tool for Speech and Voice Difficulties: Focus Group Study Among People With Parkinson Disease and Their Carers

**DOI:** 10.2196/75316

**Published:** 2025-07-16

**Authors:** Jodie Mills, Orla Duffy, Katy Pedlow, George Kernohan

**Affiliations:** 1 School of Health Sciences Faculty of Life and Health Sciences University of Ulster Belfast United Kingdom; 2 School of Nursing Faculty of Life and Health Sciences University of Ulster Belfast United Kingdom

**Keywords:** Parkinson disease, voice-assisted technology, smart speakers, speech and voice, dysarthria, speech and language therapy

## Abstract

**Background:**

People with Parkinson disease (PD) often report low volume and reduced intelligibility of speech. Common household devices that use voice-assisted technology (VAT) require users to speak slowly, clearly, and loudly for the technology to function. For people with PD, this can be challenging, but this also suggests that VAT may have potential as a therapeutic tool. While VAT is an emerging health care technology, it is important to better understand the thoughts and experiences of people with PD who are already using it despite having speech and voice difficulties.

**Objective:**

This study aimed to explore experiences of using VAT to address hypokinetic dysarthria secondary to PD, based on the perspectives of people with PD and family carers.

**Methods:**

People with PD experiencing mild to moderate speech changes who were smart speaker users, and their carers, were invited to participate in 1 of 4 in-person focus groups. Between September and December 2024, focus groups were audiovisually recorded. A semistructured topic guide informed by published evidence was used to guide discussions. Results were transcribed and analyzed through a framework analysis approach (managed using NVivo software).

**Results:**

A total of 15 participants, including 8 (53%) people with PD and 7 (47%) carers, participated in 4 in-person focus groups. Findings revealed shared experiences with VAT that were marked by its therapeutic potential and practical challenges. Five main themes were identified: (1) therapeutic potential for speech and voice, with subthemes of changes in volume, intelligibility, and clarity of speech; the role of VAT feedback; and VAT as an everyday device; (2) distrust of technology, with concerns surrounding data privacy, the listening nature of devices, and measures users take to protect themselves; (3) frustrations with devices, including devices not understanding, devices timing out, and the lack of conversation; (4) support needs, including the impact of a lack of knowledge and the need for education and guidance; and (5) design considerations for a future VAT tool in speech and language therapy (SLT).

**Conclusions:**

This study extends on previous research findings, demonstrating that VAT may be acceptable to people with PD to create changes in volume, clarity, and intelligibility. However, attention must be given to users’ privacy concerns and frustrations with devices before VAT can used as a tool in SLT. Future research should design solutions to address current usability challenges with people with PD and professionals in three ways: (1) co-designing education and guidelines for people with PD, describing the use of VAT for speech and voice difficulties; (2) refining commercial VAT for use in SLT; and (3) establishing the feasibility of a therapeutic VAT intervention for people with PD with speech and voice difficulties.

## Introduction

### Background

Parkinson disease (PD) is a progressive neurodegenerative condition that presents with rigidity, slowness of movement, and tremor [1]. In the United Kingdom, approximately 145,000 people are living with PD, including more than 4200 in Northern Ireland [2]. One of the most prevalent effects of PD is a change in speech and voice, affecting up to 90% of people with PD [3]. Also known as hypokinetic dysarthria [4], reduced volume and unclear speech worsen over time, impacting intelligibility and clarity of speech. These changes impact activity, social participation, and well-being [5].

Despite these figures, 48% of people with PD in the United Kingdom do not access speech and language therapy (SLT) [6], which may be due to long waiting lists, regional clinician shortages, and barriers related to the resource-intensive nature of therapy programs such as Lee Silverman Voice Treatment. Therefore, there is a need to explore therapy alternatives to make SLT accessible to more people with PD [7].

Voice-assisted technology (VAT) is defined as a device that uses natural language processing or automatic speech recognition (ASR) to interpret words and convert them into actionable requests. The most common commercially available VAT devices are Amazon Alexa and Google Home. Amazon Alexa is manufactured by Amazon. People with PD have been identified as a population likely to benefit from using VAT therapeutically [8]. However, VAT systems currently demonstrate poor recognition of dysarthric speech, with error rates increasing alongside speech severity [9,10]. This results in the device giving auditory or visual feedback using the recognition light, which prompts some participants with speech difficulties to change their speech to enable interaction with VAT [11-13]. Duffy et al [13] highlighted the potential of VAT to improve speech of people with PD. This study used an online survey to explore the knowledge and experiences of people with PD with VAT, in addition to reporting effects of using VAT on speech and language. In total, 290 participants reported making a deliberate effort to improve their speech clarity when interacting with VAT by specifically speaking “slower, clearer, and louder.” Similar speech changes have been reported across literature in other conditions [14,15], and this is strikingly similar to the goal of traditional SLT for people with PD. However, this survey did not solely investigate the impacts of using VAT on speech and voice in an SLT context and any reported impacts were limited by the constraints of survey methodology. Therefore, this study builds on the study by Duffy et al [13], using focus groups to provide a more in-depth and nuanced understanding of what factors contribute to this effect of increased volume and intelligibility, specifically with people with PD who use VAT regularly and report speech and voice difficulties. Focus groups may provide richer insights into experience of speech and voice difficulties, including facilitators and barriers to using VAT. Therefore, an exploration of how VAT is being used by people with PD with speech and voice difficulties and their carers is required to provide an understanding of how to use VAT to improve speech and voice outcomes as part of SLT. This ensures new technologies, such as VAT, can be used in a way that meets end user needs and may lead to a higher chance of uptake in the future [16]. An additional paper describes the perspectives of speech and language therapists regarding VAT as a tool for speech and voice difficulties in PD.

This study explores the experiences of using VAT to address hypokinetic dysarthria, secondary to PD, from the perspectives of people with PD and carers.

### Objectives

This study had the following objectives: (1) to understand the type, extent, and nature of VAT use while experiencing hypokinetic dysarthria secondary to PD; and (2) to understand experiences of those who are facilitating people with PD to use VAT with hypokinetic dysarthria.

## Methods

Qualitative research methods such as focus groups allow individuals to reflect on their own experiences in a supportive environment while also developing novel contributions though group discussions [17]. In-person focus groups were used for data collection to encourage conversations among participants and create richer data.

### Ethical Considerations

This study was reported in accordance with the Consolidated Criteria for Reporting Qualitative Research checklist [18] and is part of a larger PhD study that received ethical approval from the Ulster University Research Ethics Committee in July 2024 (approval number FCNUR-24-016-A). All participants provided informed consent. Privacy and confidentiality were ensured—only the research team were able to see personal demographic data, and data will only be held as long as the university guidelines and General Data Protection Regulation require. Following transcription, all participants’ data were anonymized. Participants were reminded that if they knew another participant at the focus group they were to respect their right to confidentiality in their participation. No participants in the research were given payment.

### Patient and Public Involvement

Before study development, a partnership was created between third-sector organization Parkinson’s UK Northern Ireland and the research team to provide a voice for people with PD and their carers throughout the research. A person with PD and a caregiver were recruited through Parkinson’s UK Northern Ireland and a speech and language therapist was recruited through research connections as “experts by experience” to co-design the research. These 3 patient and public involvement representatives influenced and informed various aspects of the study design, including participant information sheets, interview guides, and consent forms. They also contributed to the study’s conduct and dissemination through input during online meetings, advising on recruitment strategies, focus group location, and duration. Experts by experience were asked to provide feedback on information sheets via email, indicating that complex language should be avoided. Consequently, the information sheets were revised to include more patient-friendly language. Furthermore, feedback on the interview schedule was also provided, with experts by experience suggesting that focus groups should be conducted in-person rather than online, as they felt it could limit technological barriers to participation. They also suggested that introductions from researchers should include an informal preamble about smart speakers, emphasizing the technology’s relevance for people with PD and for the speech and language therapists working with this population. As a result, focus groups began with personal stories about PD and the professional journeys and provided background on what smart speakers are, why they might work to improve speech and voice, and included the lead author’s personal experience using her own Alexa device. The experts by experience offered valuable lived experience insights to the academic team during data collection and charting, including suggestions to offer the option of individual interviews to speech and language therapists who were unable to attend focus groups, to maximize data collection.

### Study Recruitment

Recruitment advertisements were posted on the research portal, featured in the Research Support Network monthly emails, and distributed as flyers to Parkinson’s Support groups local to Northern Ireland. Some of the participants had established a relationship with the lead researcher (JM) due to work with Parkinson’s UK in Northern Ireland and knew about her reasons for conducting research.

The population size could not be determined due to a lack of up-to-date data on the number of people with PD experiencing speech and voice difficulties in Northern Ireland. Therefore, 2 focus groups per each population, each comprising 3 to 4 participants, were chosen to better support people with communication disorders and provide variations in opinion while allowing data saturation. This is a smaller number than the 6 to 8 participants typically recommended in the literature, and was purposefully planned to allow discussion of ideas without discouraging quieter voices, as can happen in large groups. Participants were asked to contact the research team to express their interest in the study and were screened via telephone by the primary researcher according to predefined inclusion and exclusion criteria (Textbox 1). Potential participants were sent study information and consent forms by email or post, depending on their preference, and were asked to indicate their suitability for participation in a focus group. Once consent was gained a demographic survey, the Voice Handicap Index (VHI) [19] and a Digital Health Readiness Questionnaire was sent to participants. The VHI was included as it is a standardized tool used by speech and language therapists to measure clients’ self-reported changes in voice and is commonly used with the PD population to ensure a person-centered approach to treatment, rather than focusing solely on the disease or impairment [20]. Carers did not receive the VHI.

**Study inclusion and exclusion criteria for people with Parkinson disease (PD) and carers.**

**Inclusion Criteria for People with PD**

- Adult aged >18 years
- Mild to moderate dysarthria or voice difficulties (to include users of augmentative and alternative communication
- Diagnosis of PD
- Current or previous use of voice-assisted technology (VAT)
- Able to participate in a focus group up to 90 min

**Exclusion Criteria for People with PD**

- Moderate or severe cognitive impairment
- History of other neurological disorders

**Inclusion Criteria for Carers**

- Adult aged >18 years
- Live with or care for a person with PD
- Experience of facilitating use of VAT with a person with PD

### Procedure

Each focus group or interview was facilitated by a female speech and language therapist (JM), with a researcher present (qualified speech and language therapist or health care professional with a PhD), and lasted between 1 hour, 9 minutes, and 1 hour, 21 minutes. Focus groups were conducted in person at the Ulster University Belfast campus. A semistructured topic guide informed by literature was prepared in advance to ensure methodological consistency across focus groups, and the lead author attended focus group training before conducting the research. The topic guides were differentiated to reflect the slight differences in lived experience between people with PD and their carers when using or facilitating VAT. Prompting questions encouraged equal participant contributions and topic guides were differentiated to reflect the perspectives of each stakeholder group. This was reviewed by patient and public involvement representatives, including a person with PD, a carer, and a speech and language therapist, and refinements were made to the phrasing of questions. A summary of the questions posed to people with PD and carers is provided in Multimedia Appendices 1 and 2. Participants were invited to discuss their experiences of using VAT to address the main research question: to explore the experiences of people with PD and their carers in using VAT to address hypokinetic dysarthria. Field notes of key ideas were taken during the focus groups.

### Data Analysis

Focus groups were audiovisually recorded and transcribed using Microsoft Teams. To ensure methodological rigor and ensure contextual accuracy, transcriptions of videos were completed within 24 hours of the focus groups. All transcriptions were manually checked, and any errors were corrected before analysis. All personal and identifiable information was anonymized before analysis.

Framework analysis was used to structure the work as it enables an in-depth analysis of themes across a dataset and allowed researchers to collaborate [21]. In addition, given the lead researcher’s involvement with the Parkinson community, it allowed awareness of any biases and improved transparency [22]. The five stages of framework analysis were followed [23,24]: (1) familiarization and data immersion, (2) develop theoretical framework, (3) indexing and data charting, (4) data summary in analytical framework, and (5) data mapping and interpreting. The group discussion further refined the initial theoretical framework. The initial draft framework was developed using published evidence [8,25].

Data recordings were transcribed before being imported into qualitative data management software NVivo (version 14; Lumivero) for analysis. Following data familiarization, initial ideas were documented on a mind map with a reflexive journal by the primary researcher (JM) and shared for discussion with coauthors (GK, OD, and KP). The reflexive journal noted authors’ initial thoughts during transcription of each dataset and allowed the lead author to consider connections, similarities, and differences between data. Upon first reading, the lead author created a mind map to group these ideas together and create initial codes for analysis. Further discussion and data immersion allowed creation of draft themes, where the lead author created a second mind map considering connections between the codes, and how they could be grouped into overarching categories. Using a mind map allowed the researchers to reflect upon biases and assumptions and explore alternatives in data interpretation to ensure results accurately represented participants’ lived experiences. The framework was refined to reach consensus at further meetings with coauthors. Data indexing was completed by the primary researcher (JM). Summarization of indexed data into codes and thematic charting was completed by JM and discussed with coauthors (GK, OD, and KP) and used to formulate the final theoretical framework [21]. Original data extracts from transcriptions are included, alongside the participant number, to enhance trustworthiness and credibility of findings. Furthermore, the results were sent to all participants for member checking to ensure that the written findings reflected their lived experiences and to enhance rigor of the research. Most participants responded that they were satisfied with the reporting of research. One participant reported that they appreciated that the researchers stayed true to the “Northern Irishisms” and sentence structure used during focus groups, as this accurately represented how ideas were expressed. Changes made included checking the anonymization of quotes and incorporating headings for subthemes, as some participants felt that these changes made it easier to follow for lay readers.

## Results

### Participants

People with PD and their carers (n=16) were recruited via Parkinson’s UK in Northern Ireland, a third-sector organization. A total of 16 participants were recruited, of whom 15 (n=8, 53% people with PD and n=7, 47% carers) participated in focus groups. One carer dropped out after recruitment due to scheduling conflicts. Each group consisted of 3 to 4 participants per focus group across the 4 focus groups (Table 1). Our experience of holding separate focus groups for people with PD and their carers and not involving professionals was positive. People with PD provided open and honest reflections [21], with each contributing to the conversation, which likely would have been constrained in a mixed group. However, this resulted in a distribution of participants by sex, with mostly male people with PD and female carers. People with PD and their carers who were familiar with each other from the wider Parkinson community encouraged each other in conversations providing the richest insights and the longest focus groups. One participant in a group whose members did not know each other reported the following: “I think discussing then with each other...you learn from each other. I’ve learnt things from this group tonight.” [P5].

A total of 16 participants were recruited to the study; however, only 15 participated. People with PD (n=8) and partners or carers (n=7) attended in-person focus groups at Ulster University Belfast (Tables 2 and 3). Most of the people with PD were male (7/8, 87%) and ranged in age from 57 to 77 years. Most of them (7/8, 87%) had been diagnosed for over a year, while one had been diagnosed more recently.

**Table 1:** Format of qualitative data collection focus groups and number of participants in each group.

Population and format		Number of participants	Length of time
**People with Parkinson disease**			
	In-person focus group 1	4	1 h 21 min
	In-person focus group 2	4	1 h 12 min
**Ca** **rers or partners**			
	In-person focus group 1	3	1 h 9 min
	In-person focus group 2	4	1 h 12 min

**Table 2:** Demographic characteristics of people with Parkinson disease (PD) in in-person focus groups.

Person with PD ID	Gender	Age (y)	Year of diagnosis	Voice Handicap Index severity
P1	Male	59	2012	Moderate
P2	Male	61	2013	Normal
P3	Male	57	2023	Severe
P4	Male	73	2020	Moderate
P5	Male	77	2022	Moderate
P6	Female	70	2019	Normal
P7	Male	77	2014	Moderate
P8	Male	68	2017	Mild

**Table 3:** Demographic characteristics of carers in in-person focus groups.

Carer ID	Gender	Age (y)
P9	Female	55
P10	Female	65
P11	Female	67
P12	Male	72
P13	Female	74
P14	Female	66
P15	Female	57

The VHI is a subjective self-rating questionnaire for people with voice problems, where people with PD self-rated the impact of their voice disorder on their quality of life. The VHI uses statements about voice problems, which are ranked from 0 to 4 on frequency: 0=never, 1=almost never, 2=sometimes, 3=almost always, and 4=always. There are 10 questions across 3 sections, with 30 questions in total [19]. This covers the functional, emotional, and physical impacts of voice disorders, with a total score of 120. Scores between 0 and 30 show mild impairment, 31 to 60 moderate impairment and 60 to 120 severe impairment. On average, the participants reported a moderate impairment on the VHI [19] between 42 and 52, with 2 reporting very mild impairment (5 and 21) and one within the severe range (87).

The Digital Health Readiness Questionnaire required participants to rank 20 statements about technology use and ability using a 5-point Likert scale: 1 represented never or strongly disagree; 2 represented rarely or disagree; 3 represented sometimes or neither agree nor disagree; 4 represented often or agree; and 5 represented daily or strongly agree. Statements covered digital access, use of digital technology, digital literacy, digital health literacy, and learnability. Although 6 people with PD had good digital access, that is, they used the internet, a laptop, and a smartphone or tablet daily, only 3 used health-related applications, and the majority lacked digital health literacy. Participants had mixed digital skills. While 5 could complete basic tasks such as sending emails, sharing pictures, and video-calling, 3 indicated experiencing more difficulties. Moreover, 7 were motivated to learn about technology, and 5 agreed that digital skills could positively impact their health. Complete results from the Digital Health Readiness Questionnaire can be found in Multimedia Appendix 3.

Of the 7 carers or partners, 6 (86%) were female and 1 (14%) was male and ranged in age between 55 and 74 years. All carers had good digital access and 5 used health-related applications. Overall, carers had strong digital skills and almost all could complete basic tasks such as sending emails, sharing pictures, and video-calling confidently. All 7 carers were motivated to learn about technology and 6 agreed that digital skills could positively impact their health.

There were five main themes identified across the groups and datasets: (1) therapeutic potential for speech and voice, (2) distrust of technology, (3) frustrations with devices, (4) support needs, and (5) design considerations for a future VAT tool in SLT. Table 4 presents on overview of the themes and subthemes. The main and subthemes identified by each group are outlined with appropriate quotes in the subsequent sections to enhance credibility of results, without identifying the speaker.

**Table 4:** Results from research with themes and subthemes listed.

Themes	Subthemes
Therapeutic potential for speech and voice	Changes in volume, intelligibility, and clarity, as well as role of feedback Can act as a communication partner A useful or everyday device
Distrust of technology	Data privacy Listening nature Measures to protect themselves A more pragmatic approach
Frustrations with devices	Repetition, timing out, or error scaffolding No conversation or generalization No therapeutic relationship
Support needs	Impact of lack of knowledge Need for education
Design considerations for a future voice-assisted technology tool in speech and language therapy	Develop a speech and language therapy program Monitoring speech and voice changes Additional feedback

### Theme 1: Therapeutic Potential of VAT

Participants reported several facilitators of VAT that indicate its potential use as a clinical tool for the management of speech and voice difficulties.

#### Changes in Volume, Intelligibility, and Clarity and the Role of VAT Feedback

VAT promoted positive changes in volume, clarity, and intelligibility of speech. Positive implications for activity, participation, and well-being were also reported. Interacting with VAT caused people with PD to use adaptive strategies to change their speech.

For example, participants reported increased volume when talking to VAT:

She raises her volume and very deliberately, and says “Alexa television on.”

P12, carer

I’m a bit like you; I would shout at it. Raise my voice.

P5, person with PD

Others described increased clarity of speech and a slowing down their pace:

I would say “Alexa play me Mozart the marriage overture,” and it comes out with some pop tune, and then I’ll shout back, and then I catch myself on, and speak slowwwwly and then it would work.

P8, person with PD

I have to step back and compose myself, I suppose mentally, and speak out slower and clearer because I realised it’s my voice that’s not really getting to her.

P5, person with PD

One participant summarized their interactions with VAT as follows:

I find myself speaking much louder, slower and clearer, and then it finally pumps out the record!

P8, person with PD

The feedback provided by VAT created awareness and prompted people with PD to adapt their speech using strategies. This feedback was seen to be key to changes in volume, clarity, and intelligibility as participants indicated the following:

It doesn’t respond, maybe the first time. OK, you know that she hasn’t either heard you or you need to speak slower and louder.

P6, person with PD

You should learn if Alexa doesn’t hear you, Alexa won’t respond. You learn to repeat yourself a bit more clearly.

P12, carer

#### VAT as a Communication Partner

Carers reported that VAT could also act as a communication partner for people with PD with speech and voice difficulties, providing opportunities to rebuild confidence in using their voice:

There aren’t a huge amount of opportunities during the day for him to have a really good conversation until I come home. So... its another avenue for him to use his voice.

P9, carer

There’s a benefit, because if it’s given him more confidence to use his voice then, that’s important.

P9, carer

#### A Useful Everyday Device

In addition, people with PD and their carers reported everyday functional uses of VAT, ranging from basic requests, such as asking for the date and time, weather updates, setting reminders and timers, and playing music, to more complex requests such as finding recipes and cooking, searching for facts, checking sports scores, singing, participating in quizzes, and controlling smart home appliances.

Most participants reported using an Amazon Alexa Echo Dot device, (third or fifth generation), or a Echo Dot Kids. Only a few participants reported using a Google Home or Google Nest device. Participants owned between 1 and 3 devices in their homes, and their frequency of use varied widely from once or twice a day to more than 10 times daily. They reported having a VAT device in their kitchen, living room, or bedrooms. Playing music and listening to the radio or news were popular requests among participants in addition to medication reminders and controlling smart home appliances:

I want to get a news programme, I want music, whatever. Sit back and listen to the news or just to music and updates.

P5, person with PD

I think it’s great for the alarm because we’re both forgetting. I say ‘, Did you take your tablet?’ It’s the always forgetting.

P10, carer

It controls everything else in the house. We have to have one upstairs at night so we can get into bed at night and turn off the landing lights because the switch for the landing lights is under a picture, a big, big heavy pictures so we can’t get at it.

P14, carer

In addition, participants used their VAT devices for entertainment, for example, games and singing:

Games! You ever played games on the Alexa Party game? They all have to be speaking ones.

P2, person with PD

In addition, people with PD suggested that VAT was easy to use because of voice interaction and carers explained how this overcame the physical limitations associated with PD:

Well, I’m so used to the convenience of Alexa and not physically going and turning the radio on...You use the voice, you’ve got it.”

P8, person with PD

You can set that up where she can phone your contacts, and that’ll probably be good for my husband because he can’t use his phone. Tremor.

P10, carer

### Theme 2: Distrust of Technology

A distrust of VAT devices, with concerns surrounding data privacy, General Data Protection Regulation compliance, and governance, was reported frequently by participants. The inhuman nature of VAT, perceptions of the device as an outsider, and sometimes fear of the technology contributed to distrust. Other participants were less concerned about these risks.

#### Data Privacy

People with PD and their carers expressed caution about VAT devices eavesdropping on their conversations, as the devices are always listening. These concerns resulted from media reports and a lack of transparent communication from VAT companies regarding data privacy:

I just worry about it being a listening ear in the room. Too many people know too much about you sometimes I think.

P13, carer

Adverts start popping up and you’re going that’s spooky, because that’s what we were talking about.... They are there and they are listening, you know that.

P15, carer

#### The Listening Nature of Devices

This listening nature of devices contributed to participants’ suspicions and distrust of VAT, particularly regarding hacking:

I suppose I’m one of these people that will sit back and say how does this work. Is it all OK? Is there anything about this that’s sinister?

P11, carer

#### Protection Measures

Furthermore, privacy concerns with VAT devices meant that participants did not openly welcome devices and were distrusting, with some taking measures to protect themselves:

I still regard it as an intruder. Handle with care.

P5, person with PD

You know the way you can programme your sat NAV to have a voice from Northern Ireland? I would really like her to be one of us. I think that would just, maybe it wouldn’t feel as if it was so much of an intruder in the house.

P9, carer

I would switch it off, whenever it’s not being used. Because of this—we got a warning, we got a message from the police, Watch smart phones, watch Amazon Alexa’s because they monitor everything.

P5, person with PD

#### Pragmatic Attitudes

Despite this, some participants took a more pragmatic approach to VAT and their privacy. For example, one participant felt that VAT devices did not pick up sensitive information:

You’ve got to be sceptical to some extent of the conspiracy theorists who say that Alexa is listening to you...what are they going to do? What benefit do they get out of it?... They’re welcome to listen to our conversation! Very exciting. “You want a cup of tea?” “Yeah.” That’s it!

P12, carer

### Theme 3: Frustrations With Devices

Participants described frustrations with VAT devices, including not being understood, having to repeat themselves, devices timing out, and difficulties with error scaffolding. However, these issues did not necessarily prevent them from using the technology. Challenges with having a conversation or developing therapeutic relationship with VAT were also highlighted.

#### Frustration

Frustration when interacting with VAT was widely reported by people with PD. This was often linked to VAT not understanding people with speech and voice difficulties and them having to repeat their requests:

Hm, yeah I curse at it every time.

P4, person with PD

He’ll say something and his voice is so low, that it doesn’t catch it. And then he says it again and then he gets frustrated and he just says to me “tell that thing to do it.”

P14, carer

VAT devices often timed out before participants finished speaking, which further contributed to frustration. One person with PD reported the following:

If I do, longer sentences usually the gap in between it starts going and answering half the questions, and I go hadn’t finished yet!

P2, person with PD

Another partner described that VAT “just runs out of listening time” [P14, carer].

Feedback in this paper refers to both the blue light on the VAT devices that turns on when the device is listening, and the verbal feedback the devices may give when trying to action a request. Participants reported some difficulties taking action following VAT feedback:

And also Alexa, because I don’t know what it’s heard, it’s really hard.

P2, person with PD

He will ask her to play a song or something and she’ll play something completely different. And I say you have to speak louder or she didn’t hear you, she didn’t understand what you said.

P10, carer

#### Lack of Conversation

VAT’s limited conversational ability in standard settings was considered as unreflective of real-life communication and posed a challenge for delivering therapy. One person with PD reported the following:

To me if I’m doing anything with Alexa its either a game or your perfunctory task, you’re saying do this, do that or ring somebody. It’s not something you’re having a conversation—it’s when you get into that conversation flow, it’s trying to remember to keep your voice going sometimes.

P2, person with PD

If I’m at the kitchen sink and she’s putting stuff away with my back turned to her, you know, and she wants to say something she doesn’t want to say, “HUSBAND, I’m going to say something. Now, listen to me.” Okay? That does not happen.

P12, carer

In addition, this partner expressed skepticism of regarding VAT’s ability to help with therapy generalization:

Well, I mean if she has to raise her voice and enunciate clearly and speak slowly to Alexa, that doesn’t feed through to speaking with me.

P12, carer

#### Lack of Therapeutic Relationships

The perception that VAT might replace real speech and language therapists may be considered as a potential barrier. The therapeutic relationship developed between speech and language therapists and clients was considered missing with VAT with participants considering human clinical decision-making as an integral part of therapy:

You know you can’t be left to your machine and yes, with no humanity to it, you know? You need that person.

P11, carer

It’s face to face, it’s a person, it’s somebody that’s giving you visual feedback and giving you auditory feedback all the time and is setting the pace of the of the lesson OK. And that’s got to be important.

P12, carer

### Theme 4: Support Needs

#### Impact of a Lack of Knowledge

Participants explained that a lack of adequate knowledge about VAT capabilities and limited experimentation prevented them from fully using the potential of the devices and understanding how VAT could be a useful tool for PD:

I think there is a lot of potential in Alexa. The problem for us is, you don’t know what you can and can’t do with Alexa.

P2, person with PD

We don’t really know what he can do with it.... We’ve never done anything else with it except control the things around the house.

P14, carer

#### Need for Education and Guidance

People with PD and their partners suggested education and guidance were priorities. Suggestions included practical and technical support and layman’s explanations about how VAT use could impact speech and voice to help people with PD understand why they were using the devices during therapy:

Most importantly for us, we need to know why we’re doing it as well. I think people have Parkinson’s need to know.

P2, person with PD

I want to, I suppose, maybe to learn to use it for its available uses.

P5, person with PD

It just frightens me to go into it and start fiddling about with settings. So maybe some sort of training or help.

P13, carer

Participants discussed various methods for this education, including workshops with demonstrations, videos, reference manuals, and leaflets. Overall, it was clear that more than one delivery format of guidance would cater to varied abilities and should, “take Parkinson’s into consideration. And everyone at different level, you know, different abilities and different changes” [P13, carer].

### Theme 5: Design Considerations of a Future VAT Tool in SLT

#### Develop an SLT Program

Participants provided design considerations for the future therapeutic use of VAT for people with speech and voice difficulties in SLT, including developing an SLT “program” with interactive features, the ability to monitor speech and voice progress, and additional prompting and feedback.

Some participants embraced the idea of SLT delivered predominantly through VAT while others indicated they preferred a more hybrid approach of “speech therapist plus programme”:

If there was a built-in programme, you could interact with Alexa.

P3, person with PD

So it could be some kind of speech exercises.... Like an app even that you could put on it.

P14, carer

If there was maybe an initial speech and language session for guidance, for training and then you’re set off on your own to manage yourself.

P9, carer

Despite this, a minority of carers felt, “it is hard to see it work,” or simply preferred in person speech therapy.

Prompts and reminders could be useful to help facilitate home practice of exercises with VAT and may help people integrate everyday practice:

So he gets into the habit of remembering to take a breath, remembering just. Y’know, here’s a reminder for you, do this or do that, practice these breathing exercises in and out and projecting your voice.

P14, carer

Participants suggested numerous ideas for how an SLT Alexa skill might function or contain, including exercises, reminders to complete therapy tasks, conversation practice, feedback and prompting, and the ability to monitor progress or maintenance across tasks. For example, exercises could be delivered through an Alexa skill, which could target articulation, breath support, phonation, singing, and reading:

If your pronunciation is wrong, you look at your pronunciation before and after a game.

P1, person with PD

Maybe have Alexa read it first and then you read it. Or you read it and then have her read it back. [P9, carer]

#### Monitoring Speech and Voice Changes

The ability to track changes and measure progress in speech and voice was a common suggestion, aimed at supporting the maintenance of these skills over time:

The thing is that you’re competing against your voice of yesterday. And if you can keep it the same as your voice of yesterday, we’re ahead.

P2, person with PD

You could do, y’know your exercises at the start, and then some word work, and then go onto reading paragraphs in the second week.

P1, person with PD

Percentages and scores were suggested as feedback methods provide a measure of speech and voice when using VAT:

So if you did Alexa, and said to Alexa “Count to ten today” and it said “You counted to ten and six numbers were good, 4 numbers weren’t.”

P2, person with PD

Some people with PD described the positive impact this would have, while others felt differently, suggesting that feedback could be demotivating, counterintuitive, and negatively impact well-being:

What you would hopefully learn was, “I was better today than I was yesterday” and you’d realise where you put the emphasis or where you slowed down, and it’d guide you.

P5, person with PD

I don’t want it to be a diagnosis to say your voice is getting weaker. I want it to be, “If you keep this going for Alexa, your voice will get stronger.”

P2, person with PD

People with PD proposed using VAT’s feedback light as an alternative to scores:

Alexa is going playing the music and you’re singing along, and it goes “You’re red because you’re not high—we can’t hear you.” And then you sing high and it goes to yellow. And then you go to green. Green means your singing it properly.

P2, person with PD

A feedback feature to facilitate self-awareness of speech and voice difficulties may also be included. People with PD suggested that hearing how VAT interpreted their speech could help facilitate the use of adaptive speech strategies.

You say that, it records and sends it back, “Oh, that’s not right.” That’s what she heard, you know. That’s what it’s hearing, and then you pronounce it right.

P1, person with PD

And the programme would go, “You’re going too fast. You’re going too slow. I don’t understand.” Yeah. So just give you feedback because see, once you’ve got feedback you can go, ok right it says slow down. So you go sloooower.

P2, person with PD

In addition, more specific feedback from VAT on speech and voice was suggested:

If it could interact with you and say you’re not speaking clearly enough. I’m speaking at it and “You’re not clear enough, you’re better now.”

P5, person with PD

## Discussion

### Principal Findings

The aim of this study was to explore the experiences of using VAT to address hypokinetic dysarthria, secondary to PD, from a range of stakeholder perspectives. People with PD and their carers discussed the therapeutic potential of VAT for speech and voice difficulties, with it being regarded as a novel technology that could promote louder speech with increased clarity and intelligibility. However, challenges such as frustrations with devices and distrust of technology must be addressed. By meeting participants’ support needs and integrating feasible design considerations, the use of VAT as a tool in SLT may be considered in future research.

### Evaluating the Therapeutic Potential of VAT

People with PD reported that using VAT made them speak clearer and raise their volume. This indicates that VAT can facilitate unlimited practice attempts and prompt participants to adapt their speech [14,15,26], resulting in a therapeutic benefit. These findings build on existing research that highlights VAT’s potential role in improving and maintaining speech volume, clarity, and intelligibility [8,11,13,25] and the role of VAT feedback in shaping improvements in self-awareness, which led to self-reported improvements. In addition, VAT could facilitate practice of articulation, clarity, and volume exercises—the speech subsystems commonly targeted by speech and language therapists during therapeutic management of PD [27]. Thus, VAT could maximize therapeutic outcomes, while reducing health care resource demands associated with intensive interventions such as Lee Silverman Voice Treatment.

While this research focused on people with PD, previous research has also emphasized a gap in knowledge regarding potential for VAT as a therapeutic support for people with motor neuron disease with speech and voice difficulties [12]. Given the aforementioned findings, this warrants further research into therapeutic potential of VAT for neurodegenerative conditions as a tool for speech and voice difficulties. This study builds upon the findings by Duffy et al [13] to inform the design of a feasibility trial that meets the needs of users and integrates use solutions current difficulties. To inform practice, future research should consider the studies by Quinn et al [28], who used interviews and device logs to evaluate the acceptability and feasibility of a smart speaker–delivered physical activity program, and Smith et al [10] and Makin et al [29] who used before and after measures to examine if a VAT intervention could improve speech clarity for adults with intellectual disability. Future research may seek to extend the work by Smith et al [10] with people with PD, focusing on volume, clarity, and intelligibility with before and after measures.

Although VAT has therapeutic potential, there are potential caveats that require further exploration. ASR models, which underpin VAT, can be variable in their understanding of speech for reasons other than speech clarity, volume, and intelligibility [30]. For example, device updates, user ethnicity, and regional accents can impact word error rates. Indeed, research indicates that black speakers had a higher word error rate than white speakers when using common commercial VAT systems such as Apple, Amazon, and Google [31]. In addition, VAT users may adapt nonstandard accents to facilitate interactions with VAT [32], which is likely to have implications for users from ethnic minority backgrounds or with regional accents. People who use VAT might change their accents, if they do not have a standard English accent, as well as increasing their volume and speaking more clearly. Therefore, users should be careful that they do not begin to use westernized accents in favor of regional or international accents when using VAT to help the device to understand them better.

The wider literature has also indicated that VATs’ limited conversational abilities was a usability issue [33]. It may be suggested that the transactional nature of VAT requests means that using VAT to practice speech may not carry over into real-life conversations [30]. While VAT may be useful to isolate practice of impaired volume and intelligibility across word, phrase, and sentence level, it may be difficult to generalize practice into conversation and create meaningful change in activity or participation. Furthermore, research investigating the error rates of ASR, which underpins VAT, has shown that error rates are higher for conversational speech than for short phrases or read speech in people with speech disorders [34,35]. Therefore, it is difficult to know if VAT could facilitate meaningful conversation practice for people with PD that generates increased real-life conversational participation. Future research should seek to establish how features of commercial VAT could be used to promote more conversational engagement with VAT for people with PD. This would build on the current research, solving the reported barrier of a lack of conversational engagement with VAT, and may enable conversational practice with VAT in the context of SLT.

### Turning Distrust of VAT Into Reassurance

Concerns for data privacy, security, and unwanted surveillance are well documented in relation to VAT use among older adults and in therapeutic interventions [8,28,36]. Previous research with people with PD indicated that between 9% and 33% of people expressed privacy concerns regarding VAT [8]. Similar concerns emerged in this study, potentially influenced by significant and rising media discourse about artificial intelligence in the media [37], and perceptions of VAT as an artificial intelligence device. However, other factors may contribute, such as varying levels of digital literacy, as reported among people with PD and their carers in this study, and less frequent use of VAT, which for some resulted in fear toward technology—a pattern observed with the older population [38]. Others were actively embracing VAT, potentially due to a high level of digital capabilities, personal innovation, habit, positive attitudes to technology, or less aversion to risk [39]. While privacy pragmatism and cynicism concur with wider literature [40], other people with PD have previously reported that they are actively embracing new technological advances [8] with high levels of ownership and use of VAT reported among older adults. This builds on previous research [8] by highlighting the role of digital skills and habit in shaping privacy concerns and trust in technology and emphasizing the need for digital literacy education. Future research may build on current findings by collaborating with people with PD and their carers to discuss the best delivery method for digital skill upskilling.

Despite these concerns, privacy concerns alone do not deter people with PD and their carers from using VAT. Efforts should be made to upskill the digital skills of people with PD, increase their knowledge of privacy protection, and promote active security measures to mitigate risks. This concurs with results from the Digital Health Readiness Questionnaire, where people with PD lacked digital literacy and had mixed digital skillsets, but were motivated to learn about technology. Although efforts may also upskill carers, participants in this research had strong digital skills and literacy. Future research should seek to reassure people with PD and their partners about the integrity of VAT’s privacy policies and surveillance concerns and develop tailored guidance to support users with diverse digital skill levels and age groups. This would build on the current research by using specific privacy concerns and providing solutions that directly address user needs and may positively impact use.

### Navigating Frustrations With VAT

Technological limitations of VAT and resulting frustrations were discussed, including issues with VAT not understanding speech; devices timing out; and a lack of meaningful feedback, conversation, and relationship. Similar frustrations surrounding the inconsistency of VAT have been reported by people with dysfluent speech [41] and neurological disabilities in wider research, particularly where VAT did not understand them, or provide meaningful feedback on errors [42].

In addition, VAT should be used cautiously for feedback on speech and voice due to potential well-being implications and users should be careful not to assign responsibility for all VAT inaccuracies to speech and voice [30]. Speakers can blame themselves for ASR errors and link this to their sense of identity, including racial, regional, and location identity. Therefore, participants may modify their dialect and word choice to be understood by ASR [32], which may negatively impact their sense of self. Despite acknowledged limitations, subjective improvements in confidence, self-awareness, accessibility, and well-being have been reported when using VAT. Wider literature has also demonstrated increased independence, well-being, and quality of life for older adults following the use of VAT [43]. This paper provides the foundations for future research to cocreate solutions to the use barriers identified by people with PD and their carers. By using ideation methodology, such as design thinking, and participatory design methods, future research could integrate multistakeholder collaboration to create solutions that are rooted in user needs, as identified by this study.

### Supporting Adoption and Use of VAT

Although people with PD and their carers were at varying stages on their journey with VAT adoption, increased use generally corresponded with greater integration into their daily routines. This pattern mirrors broader trends observed in the general population [44]. However, participants frequently reported a lack of awareness of VAT’s full functionality and capabilities, which they identified as a barrier to wider adoption. Given that smart speakers ownership approximately doubled during the pandemic, from 22% of households in 2020% to 39% [44], this knowledge gap is somewhat unexpected.

The level of VAT use and integration by participants appeared to shape their attitudes toward its usefulness and ease of use. This aligns with established technology adoption models, such as the Unified Theory of Acceptance and Use of Technology 2 (UTAUT2) [45], which suggest that factors such as performance expectancy (perceived usefulness) and effort expectancy (ease of use) can impact technology acceptance. In this context, people with PD who perceived VAT as beneficial and easy to use were more likely to integrate it into their routines. This highlights that future refinement of a VAT tool should aim to show why VAT is easy to use and useful for speech and voice to increase adoption by people with PD. However, those unfamiliar with VATs’ capabilities struggled to extrapolate their everyday uses of VAT to speech and voice therapy. Furthermore, the privacy-personalization paradox [46] offers insights into VAT adoption, as it suggests that while users appreciate that personalized technology can enhance their everyday lives, concerns about data privacy can create hesitation around its use. Indeed, some people with PD and their carers reported reluctance to use VAT due to uncertainty about data privacy and security. Addressing these concerns through education and structured guidance on VAT’s therapeutic applications could facilitate greater adoption and integration into SLT practices and extend on this study’s findings.

In addition, participants indicated that they would benefit from education and training. This reflects findings from previous research surrounding therapeutic use of VAT in SLT and with older adults [25,36], indicating that digital health interventions should include education in how to use technology effectively and provide technical support throughout. While Cave [30] recommended development of a clear and accessible guide to using ASR tools, no research to date has created or examined an educational intervention for VAT as a therapeutic tool for speech and voice difficulties [47]. Future research may use facilitators and barriers to VAT use, identified by this study, to cocreate training and educational resources for people with PD and their carers, by using solution focused idea generation workshops and integrating behavior change frameworks [48]. This may also include identification of best methods for delivering training effectively to encourage increased confidence, adoption, and use of VAT for speech and voice difficulties.

### Design Considerations for a Future VAT Tool

Participants in the study made various suggestions about a future VAT tool for therapeutic management of speech and voice, including a specific skill for VAT, extended feedback on speech and voice, and the ability to measure progress. Esquivel et al [42] indicated similar suggestions for use of VAT for people with neurological disabilities, including a voice assistant hub with suggested skills, training, and a repository with personal stories. Duffy et al [13] and Kulkarni et al [25] also suggested that additional research may include user experience in a therapeutic VAT tool. Wider research advocates for including the lived experiences of people with PD using VAT for speech and voice in the potential design, development, and testing of VAT tools [13,42].

Therefore, future research should build upon these identified uses, facilitators, and barriers, using participatory co-design methods with speech and language therapists, people with PD, carers, and technology experts to brainstorm solutions to some of these perceived barriers with VAT. Co-design is an established way to develop health care technology and supports people with communication difficulties [49]. This ensures that VAT reflects the needs of people with PD and their carers, reducing abandonment of VAT and digital exclusion for people with PD [29].

### Limitations

This study was conducted exclusively with people with PD and their carers in Northern Ireland. While this may be identified as a limitation, it is likely that their views are representative of experiences of people with PD across the United Kingdom and beyond with some variation in technology adoption globally. Technology use and adoption varies across countries and the findings may not represent the global experiences of people with PD who are VAT users. Furthermore, the sample of people with PD recruited in this study were White. This poses a question of how results may vary with potential cultural and regional differences. Future studies should seek to replicate findings to understand the generalizability of results, recruiting a range of people with PD from diverse cultural and ethnic backgrounds.

Inclusion in the study required participants to be actively engaging with VAT. Therefore, this indicates that people with PD and their carers were self-selecting participants and may have already had the basic digital skills that facilitated the adoption of VAT. As such, they may have been biased toward the use of technology and have a positive outlook toward its use. This is a limitation in any qualitative work that explores a particular subject. Other people with PD and their carers who may not have adequate digital skills may have had a different outlook and a less favorable attitude toward VAT use.

### Recommendations

Our study highlights several suggestions for future research, as outlined in the Discussion section. The following recommendations are suggested to develop the therapeutic use of VAT:

1. Refinement of commercial VAT, specifically for targeting volume, clarity, and intelligibility of speech as part of SLT, using user-led co-design: this may include feasible solutions to technological limitations, which do not redevelop the technology, and extended conversational abilities, which meet the needs of people with PD and their carers and improving their experience.
2. Co-design of clear and accessible education and guidelines to help meet the needs of people with PD and their carers: this may include realistic expectations of how VAT can benefit speech and voice, a summary of VAT’s capabilities and information on privacy. Future work must also identify the optimal route for delivery of education and training, particularly for those who lack basic digital skills.
3. Establishing the feasibility of a therapeutic VAT intervention for people with PD with speech and voice difficulties, including pre- and postacoustic analysis of voice or self-reported impacts in volume, clarity, and intelligibility.

### Conclusions

This study builds upon the findings of Duffy et al [13] by exploring the experiences of people with PD and their carers using VAT with speech and voice difficulties. Participants reported positive therapeutic changes in volume, clarity, and intelligibility; however, frustrations with technology and privacy concerns may need to be addressed before VAT can be integrated into SLT. The next step will explore the role of multistakeholder collaboration to co-design feasible solutions to VATs’ usability challenges with people with PD, their carers, speech and language therapists, and technology experts. Furthermore, additional work is needed to trial commercially available VATs as a tool for improving speech and communication changes in people with PD, as well as to explore potential generalization and maintenance effects.

## Appendix

Multimedia Appendix 1 Focus group discussion guide for people with Parkinson disease.

Multimedia Appendix 2 Focus group discussion guide for carers.

Multimedia Appendix 3 Digital Health Readiness Questionnaire results.

## Abbreviations

ASR: automatic speech recognition
PD: Parkinson disease
UTAUT: Unified Theory of Acceptance and Use of Technology 2
VAT: voice-assisted technology
VHI: Voice Handicap Index
