# Supplementary figures and images for: Exploring the Perceptions of Voice-Assisted Technology as a Tool for Speech and Voice Difficulties: Focus Group Study Among People With Parkinson Disease and Their Carers
Source: JMIR Rehabil Assist Technol. 2025 Jul 16;12:e75316. doi: 10.2196/75316 (PMC12311396; doi:10.2196/75316)

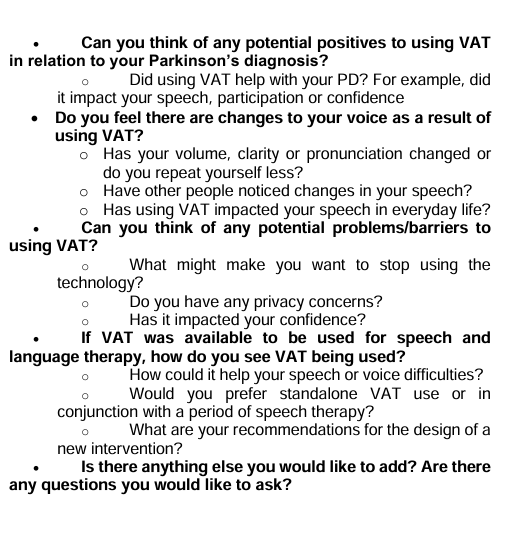

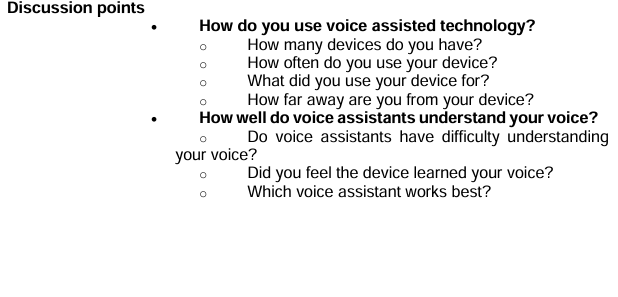

Supplement: Multimedia Appendix 1 [file rehab_v12i1e75316_app1.docx]

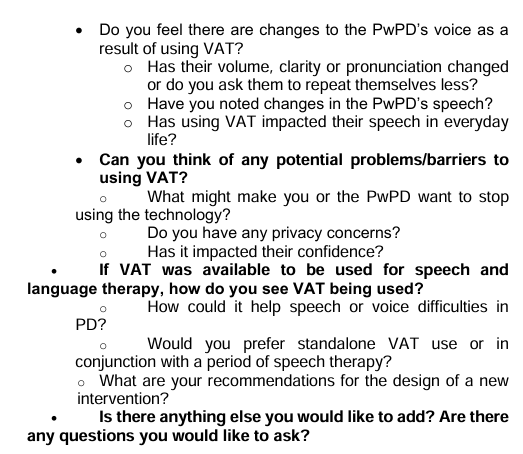

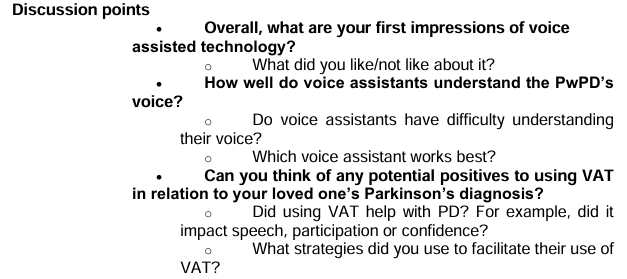

Supplement: Multimedia Appendix 2 [file rehab_v12i1e75316_app2.docx]
