# Supplementary material for: Exploring the Perceptions of Voice-Assisted Technology as a Tool for Speech and Voice Difficulties: Focus Group Study Among People With Parkinson Disease and Their Carers
Source: JMIR Rehabil Assist Technol. 2025 Jul 16;12:e75316. doi: 10.2196/75316 (PMC12311396; doi:10.2196/75316)
Supplement: Multimedia Appendix 3 [file rehab_v12i1e75316_app3.docx]

**Appendix 3 – Digital Health Readiness Questionnaire results for people with Parkinson’s and carers**

| **Digital Access (people with Parkinson’s)** | I use the internet | I use a computer or laptop | I use a smartphone and or tablet | I use a wearable (fitness tracker, smartwatch etc) |
| --- | --- | --- | --- | --- |
| P1 | 5 - daily | 5 - daily | 5 - daily | 5 - daily |
| P2 | 5 - daily | 5 - daily | 5 - daily | 2 - rarely |
| P3 | 5 - daily | 2 - rarely | 5 - daily | 5 - daily |
| P4 | 2 - rarely | 2 - rarely | 3 - sometimes | 5 - daily |
| P5 | 5 - daily | 5 - daily | 5 - daily | 1 - never / I don't have any wearable |
| P6 | 5 - daily | 5 - daily | 5 - daily | 5 - daily |
| P7 | 3 - Sometimes | 1 - never | 5 - daily | 1 - never / I don't have any wearable |
| P8 | 5 - daily | 5 - daily | 5 - daily | 1 - never / I don't have any wearable |

| **Digital Access (carers)** | I use the internet | I use a computer or laptop | I use a smartphone and or tablet | I use a wearable (fitness tracker, smartwatch etc) |
| --- | --- | --- | --- | --- |
| P9 | 5 - daily | 5 - daily | 5 - daily | 1 - never / I don't any wearable |
| P10 | 5 - daily | 4 - often | 5 - daily | 5 - daily |
| P11 | 5 - daily | 5 - daily | 5 - daily | 1 - never / I don't any wearable |
| P12 | 5 - daily | 5 - daily | 5 - daily | 1 - never / I don't any wearable |
| P13 | 5 - daily | 5 - daily | 5 - daily | 5 - daily |
| P14 | 5 - daily | 4 - often | 5 - daily | 5 - daily |
| P15 | 5 - daily | 5 - daily | 5 - daily | 5 - daily |

| **Usage of digital technology (people with Parkinson’s)** | I am able to write and send an email independently | I use social media | I am able to perform videocalling | I am able to take a picture and send it to another person | I am able to register and review my daily step count |
| --- | --- | --- | --- | --- | --- |
| P1 | 5 - strongly agree | 5 - daily | 5 - strongly agree | 5 - strongly agree | 5 - strongly agree |
| P2 | 5 - strongly agree | 5 - daily | 5 - strongly agree | 5 - strongly agree | 1 - strongly disagree |
| P3 | 5 - strongly gree | 5 - daily | 5 - strongly agree | 5 - strongly agree | 5 - strongly agree |
| P4 | 1 - strongly disagree | 1 - no | 4 - agree | 3 - neither agree nor disagree | 4 - agree |
| P5 | 5 - strongly agree | 1 - no | 5 - strongly agree | 5 - strongly agree | 5 - strongly agree |
| P6 | 5 - strongly agree | 5 - strongly agree | 5 - strongly agree | 5 - strongly agree | 5 - strongly agree |
| P7 | 1 - strongly disagree | 1 - strongly disagree | 1 - strongly disagree | 2 - disagree | 1 - strongly disagree |
| P8 | 5 - strongly agree | 1 - no | 3 - neither agree nor disagree | 5 - strongly agree | 5 - strongly agree |

| **Usage of digital technology (carers)** | I am able to write and send an email independently | I use social media | I am able to perform videocalling | I am able to take a picture and send it to another person | I am able to register and review my daily step count |
| --- | --- | --- | --- | --- | --- |
| P9 | 5 - strongly agree | 5 - daily | 5 - strongly agree | 5 - strongly agree | 5 - strongly agree |
| P10 | 5 - strongly agree | 5 - daily | 5 - strongly agree | 5 - strongly agree | 5 - strongly agree |
| P11 | 5 - strongly agree | 3 - sometimes | 4 - agree | 4 - agree | 4 - agree |
| P12 | 5 - strongly agree | 4 - agree | 5 - strongly agree | 5 - strongly agree | 3 - neither agree nor disagree |
| P13 | 5 - strongly agree | 3 - sometimes | 3 - neither agree nor disagree | 4 - agree | 4 - agree |
| P14 | 5 - strongly agree | 5 - daily | 5 - strongly agree | 5 - strongly agree | 5 - strongly agree |
| P15 | 5 - strongly agree | 5 - daily | 5 - strongly agree | 5 - strongly agree | 5 - strongly agree |

| **Digital Literacy (people with Parkinson’s)** | I know how to find helpful and reliable information on the internet | I feel safe when looking up information on the internet | I feel in control when looking up information on the internet |
| --- | --- | --- | --- |
| P1 | 5 - strongly agree | 5 - strongly agree | 5 - strongly agree |
| P2 | 5 - strongly agree | 5 - strongly agree | 5 - strongly agree |
| P3 | 5 - strongly agree | 5 - strongly agree | 5 - strongly agree |
| P4 | 4 - agree | 3 - neither agree nor disagree | 3 - neither agree nor disagree |
| P5 | 5 - strongly agree | 5 - strongly agree | 5 - strongly agree |
| P6 | 5 - strongly agree | 5 - strongly agree | 5 - strongly agree |
| P7 | 1 - strongly disagree | 2 - disagree | 2 - disagree |
| P8 | 4 - agree | 5 - strongly agree | 5 - strongly agree |
| **Digital Literacy (carers)** | I know how to find helpful and reliable information on the internet | I feel safe when looking up information on the internet | I feel in control when looking up information on the internet |
| P9 | 5 - strongly agree | 5 - strongly agree | 5 - strongly agree |
| P10 | 5 - strongly agree | 5 - strongly agree | 5 - strongly agree |
| P11 | 4 - agree | 4 - agree | 4 - agree |
| P12 | 5 - strongly agree | 5 - strongly agree | 5 - strongly agree |
| P13 | 4 - agree | 3 - neither agree nor disagree | 3 - neither agree nor disagree |
| P14 | 5 - strongly agree | 5 - strongly agree | 5 - strongly agree |
| P15 | 5 - strongly agree | 5 - strongly agree | 5 - strongly agree |
| P16 |  |  |  |

| **Digital Health Literacy (people with Parkinson’s)** | I use the internet to find more information about my symptoms, health status or medication | I use health-realted applications to follow up my health status | I am able to identify trustworthy, reliable health information on the internet |
| --- | --- | --- | --- |
| P1 | 3 - neither agree nor disagree | 1 - strongly disagree | 5 - strongly agree |
| P2 | 3 - neither agree nor disagree | 2 - disagree | 4 - agree |
| P3 | 5 - strongly agree | 5 - strongly agree | 5 - strongly agree |
| P4 | 2 - disagree | 2 - disagree | 2 - disagree |
| P5 | 5 - strongly agree | 5 - strongly agree | 4 - agree |
| P6 | 5 - strongly agree | 5 - strongly agree | 5 - strongly agree |
| P7 | 1 - strongly disagree | 2 - disagree | 1 - strongly disagree |
| P8 | 5 - strongly agree | 3 - neither agree nor disagree | 5 - strongly agree |
| **Digital Health Literacy (carer)** | I use the internet to find more information about my symptoms, health status or medication | I use health-realted applications to follow up my health status | I am able to identify trustworthy, reliable health information on the internet |
| P9 | 5 - strongly agree | 3 - neither agree nor disagree | 5 - strongly agree |
| P10 | 5 - strongly agree | 5 - strongly agree | 5 - strongly agree |
| P11 | 4 - agree | 4 - agree | 5 - strongly agree |
| P12 | 5 - strongly agree | 3 - neither agree nor disagree | 5 - strongly agree |
| P13 | 4 - agree | 4 -agree | 3 - neither agree nor disagree |
| P14 | 5 - strongly agree | 5 - strongly agree | 5 - strongly agree |
| P15 | 5 - strongly agree | 5 - strongly agree | 5 - strongly agree |

| **Learnability (people with Parkinson’s)** | I am motivated to learn more about digital technology and how to use it myself | I feel confident that I can learn about digital technology and how to use it myself | I believe that I will learn quickly when offered written information about digital technology | I believe that I will learn quickly when offered personal guidance about digital technology | I expect that learning digital skills can positively impact my health |
| --- | --- | --- | --- | --- | --- |
| P1 | 5 - strongly agree | 5 - strongly agree | 5 - strongly agree | 5 - strongly agree | 3 - neither agree nor disagree |
| P2 | 4 - agree | 4 - sgree | 4 - agree | 4 - agree | 4 - agree |
| P3 | 4 - agree | 4 - agree | 4 - agree | 4 - agree | 5 - strongly agree |
| P4 | 4 - agree | 4 - agree | 3 - neither agree nor disagree | 4 - agree | 4 - agree |
| P5 | 5 - strongly agree | 5 - strongly agree | 3 - neither agree nor disagree | 4 - agree | 3 - neither agree nor disagree |
| P6 | 5 - strongly agree | 5 - strongly agree | 5 - strongly agree | 5 - strongly agree | 5 - strongly agree |
| P7 | 3 - Neither agree nor disagree | 3 - Neither agree nor disagree | 3 - Neither agree nor disagree | 2 - disagree | 2 - disagree |
| P8 | 4 - agree | 4 - agree | 5 - strongly agree | 4 - agree | 5 - strongly agree |

| **Learnability (carers)** | I am motivated to learn more about digital technology and how to use it myself | I feel confident that I can learn about digital technology and how to use it myself | I believe that I will learn quickly when offered written information about digital technology | I believe that I will learn quickly when offered personal guidance about digital technology | I expect that learning digital skills can positively impact my health |
| --- | --- | --- | --- | --- | --- |
| P9 | 5 - strongly agree | 5 - strongly agree | 5 - strongly agree | 5 - strongly agree | 5 - strongly agree |
| P10 | 5 - strongly agree | 5 - strongly agree | 4 - agree | 4 - agree | 4 - agree |
| P11 | 4 - agree | 4 - agree | 4 - agree | 4 - agree | 4 - agree |
| P12 | 5 - strongly agree | 5 - strongly agree | 5 - strongly agree | 5 - strongly agree | 4 - agree |
| P13 | 4 - agree | 4 - agree | 4 - agree | 4 - agree | 3 - neither agree nor disagree |
| P14 | 5 - strongly agree | 5 - strongly agree | 5 - strongly agree | 5 - strongly agree | 5 - strongly agree |
| P15 | 5 - strongly agree | 5 - strongly agree | 5 - strongly agree | 5 - strongly agree | 5 - strongly agree |
